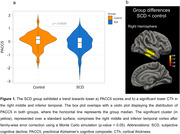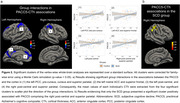# Exploring PACC5 Associations with Cortical Thickness in Individuals with Subjective Cognitive Decline

**DOI:** 10.1002/alz.091218

**Published:** 2025-01-03

**Authors:** Lídia Mulet‐Pons, Lídia Vaqué‐Alcázar, Cristina Solé‐Padullés, María Cabello‐Toscano, Oriol Perera‐Cruz, Kilian Abellaneda‐Pérez, Gabriele Cattaneo, Javier Solana Sánchez, Vanessa Alviarez‐Schulze, Josep Mª Tormos‐Muñoz, Alvaro Pascual‐Leone, David Bartrés‐Faz

**Affiliations:** ^1^ Department of Medicine, Faculty of Medicine and Health Sciences, Institute of Neurosciences, University of Barcelona, Barcelona Spain; ^2^ Institute of Biomedical Research August Pi i Sunyer (IDIBAPS), Barcelona Spain; ^3^ Sant Pau Memory Unit, Hospital de la Santa Creu i Sant Pau, Biomedical Research Institute Sant Pau, Universitat Autònoma de Barcelona, Barcelona Spain; ^4^ Department of Medicine, Faculty of Medicine and Health Sciences, Institute of Neurosciences, University of Barcelona, Barcelona, Spain. Institut d’Investigacions Biomèdiques August Pi i Sunyer (IDIBAPS), Barcelona Spain; ^5^ Institut Guttmann, Institut Universitari de Neurorehabilitació adscrit a la Universitat Autònoma de Barcelona, Badalona, Barcelona Spain; ^6^ Fundació Institut d'Investigació en Ciències de la Salut Germans Trias i Pujol, Badalona Spain; ^7^ Centro de Investigación Translacional San Alberto Magno, Facultad Ciencias de la Salud, Universidad Católica de Valencia, Valencia Spain; ^8^ Hinda and Arthur Marcus Institute for Aging Research and Deanna and Sidney Wolk Center for Memory Health, Hebrew SeniorLife, Boston, MA USA; ^9^ Department of Neurology, Harvard Medical School, Boston, MA USA

## Abstract

**Background:**

Individuals with subjective cognitive decline (SCD) express concern about self‐perceived cognitive decline, despite no objective impairment, and are at higher risk of developing Alzheimer’s disease (AD). The preclinical Alzheimer’s cognitive composite (PACC5) is a sensitive cognitive marker frequently used in preclinical AD, delineating cognitive trajectories based on amyloid status in SCD. The relationship between PACC5 and brain structure remains unexplored. We aimed to study whether PACC5 associations with brain structure in an SCD group differ from those in a control group.

**Method:**

Six hundred sixteen participants from the Barcelona Brain Health Initiative cohort (https://bbhi.cat/en) underwent magnetic resonance imaging acquisition and neuropsychological assessment. Among them, 89 healthy participants (56.18±7.72 years, 61 females) met the SCD criteria (memory concerns within the last 2 years). Meanwhile, 89 non‐SCD participants were age, sex, and educationally matched as the control group. PACC5 included z‐scores from dementia screening, episodic memory, processing speed, and category fluency tests. Cortical thickness (CTh) maps were generated with Freesurfer using T1‐ and T2‐weighted images. Whole‐brain vertex‐wise general linear models were employed and adjusted by age, sex, and educational level.

**Result:**

The SCD group showed a trend toward lower PACC5 scores (W = 4569.5, p = 0.055; Figure 1a) and a significantly thinner cortex in the right middle and inferior temporal (CWP = 0.00898; Figure 1b). Examining PACC5‐CTh associations, group interactions emerged in four clusters (Figure 2a): the left posterior cingulate cortex (CC) and precuneus (CWP = 0.005), the left rostral anterior CC and superior frontal (CWP = 0.012), and the left and right post‐central cortex (CWP = 0.019, 0.001, respectively). Separate examinations uncovered a positive association in the right post‐central and superior parietal exclusively for the SCD group (CWP = 0.001; Figure 2b).

**Conclusion:**

Adults with SCD trend toward a lower PACC5, presenting a reduced temporal CTh, an early atrophied region reported in neurodegenerative disorders, compared to the controls. Thinner left anterior and posterior CC and precuneus contributed to the lower PACC5 in SCD compared to controls. These results are relevant, as the CC role in resilience processes for cognitive impairment in AD is proposed, an aspect underexplored in SCD.